# Technologies used in the treatment of burn victims in intensive care: a scope review

**DOI:** 10.1590/0034-7167-2022-0738

**Published:** 2024-05-13

**Authors:** Kyohana Matos de Freitas Clementino, Gabriela Duarte Bezerra, Gleice Adriana Araujo Gonçalves, Maria Corina Amaral Viana, Luis Rafael Leite Sampaio, Woneska Rodrigues Pinheiro

**Affiliations:** I Universidade Regional do Cariri. Crato, Ceará, Brazil

**Keywords:** Burns, Intensive Care Unit, Therapy, Technology, Nursing Care, Quemaduras, Unidades de Cuidados Intensivos, Terapéutica, Tecnología, Atención de Enfermería, Queimaduras, Unidade de Terapia Intensiva, Terapêutica, Tecnologia, Cuidados de Enfermagem

## Abstract

**Objectives:**

to analyze the technologies used by the nursing team in the treatment of skin lesions caused by burns in patients under intensive care.

**Methods:**

this is a scope review conducted on the LILACS, Medline, PubMed, and CINAHL databases without temporal or language restrictions.

**Results:**

the highlighted technologies included the use of specialized dressings, biological agents such as probiotics and cyanobacteria, as well as negative pressure therapies and enzymes such as papain and collagenase. Some technologies, such as nanocrystalline silver, demonstrated efficacy in infection control.

**Final Considerations:**

the study identified essential technologies in burn care, emphasizing the need for further research on “soft” technologies. The findings support the promotion of evidence-based nursing care for burn patients in intensive care and enhance knowledge about effective treatments.

## RESUMO

**Objetivos:**

analisar as tecnologias utilizadas pela equipe de enfermagem no tratamento de lesões cutâneas causadas por queimaduras em pacientes sob cuidados intensivos.

**Métodos:**

trata-se de uma revisão de escopo realizada nas bases LILACS, Medline, PubMed e CINAHL sem restrição temporal ou de idiomas.

**Resultados:**

as tecnologias destacadas abrangeram o uso de coberturas especializadas, agentes biológicos como probióticos e cianobactérias, além de terapias de pressão negativa e enzimas como a papaína e colagenase. Algumas tecnologias, como a prata nanocristalina, mostraram eficácia no controle de infecções.

**Considerações Finais:**

o estudo identificou as tecnologias essenciais na assistência às queimaduras, enfatizando a necessidade de pesquisas adicionais sobre tecnologias “leves”. As descobertas sustentam a promoção de uma assistência de enfermagem baseada em evidências para pacientes queimados sob cuidados intensivos e aprimoram o conhecimento sobre tratamentos eficazes.

## RESUMEN

**Objetivos:**

analizar las tecnologías utilizadas por el equipo de enfermería en el tratamiento de lesiones cutáneas causadas por quemaduras en pacientes en cuidados intensivos.

**Métodos:**

se trata de una revisión de alcance realizada en las bases de datos LILACS, Medline, PubMed y CINAHL sin restricciones temporales ni de idioma.

**Resultados:**

las tecnologías destacadas incluyeron el uso de coberturas especializadas, agentes biológicos como probióticos y cianobacterias, además de terapias de presión negativa y enzimas como la papaína y la colagenasa. Algunas tecnologías, como la plata nanocristalina, mostraron eficacia en el control de infecciones.

**Consideraciones Finales:**

el estudio identificó las tecnologías esenciales en la atención de quemaduras, enfatizando la necesidad de investigaciones adicionales sobre tecnologías “leves”. Los hallazgos respaldan la promoción de una atención de enfermería basada en evidencia para pacientes quemados en cuidados intensivos y mejoran el conocimiento sobre tratamientos efectivos.

## INTRODUCTION

Burn injuries, resulting from thermal, chemical, or electrical agents, occur when excessive heat damages body tissues. These injuries can be classified based on the depth of the affected layers, correlating with patient morbidity, and are categorized as superficial, superficial partial, deep partial, and full-thickness burns^(1)^.

Estimates indicate that out of one million burn accidents in the country, only 10% of those involved seek hospital treatment, and a total of 2,500 victims die due to these injuries. Children under 15 and the elderly in a home environment are the most frequent victims of this type of accident^(2)^.

Several factors must be considered when dealing with a burn victim and influence treatment decisions, including the depth, extent, and location of the burn; the victim’s age; pre-existing conditions; aggravating factors; and smoke inhalation. The rule of nines is used to accurately assess the extent of a burn, estimating that the head and neck, as well as each upper limb, correspond to 9%; each lower limb to 18%; and the chest to 36% of the body^(2)^.

Nursing care for burn patients is complex, ranging from initial neurological assessment to care specific to each type of injury. Therefore, a technical-scientific knowledge base is required to support the professional in their practice. The first step involves maintaining airway patency, fluid replacement, and pain control. The nurse is also responsible for monitoring and controlling the saturation of patients with spontaneous breathing or on oxygen therapy, as well as establishing two large-caliber peripheral venous accesses for fluid replacement^(2)^.

Burn patients admitted to the Intensive Care Unit (ICU) are categorized as major burn victims if they have second-degree burns on more than 20% of the burned body surface area (TBSA) and third-degree burns on more than 10% of the TBSA; perineal burns; third-degree burns on hands, feet, face, neck, or armpits; or burns caused by electric shock^(3)^.

Also considered major burn patients are those who have suffered burns associated with the following conditions: inhalation injury, polytrauma, head trauma, shock of any origin, renal insufficiency, heart failure, liver failure, diabetes, hemostasis coagulation disorders, pulmonary embolism, acute myocardial infarction, severe infectious conditions resulting from burns or not, compartmental syndrome, consumptive diseases, or any other condition that could complicate the burn^(3)^.

Performing dressings on burn injuries is essential for wound cleaning, stimulating the healing process, preventing infection, and reducing pain associated with the injury. To collectively achieve these goals, technologies in the field of dressings and wound care should be used, considering individualized care and analyzing the biopsychosocial aspects of the patient^(4)^.

Thus, the concept of technology can be perceived in two ways: as a process, i.e., the creation and structure of didactic-pedagogical materials, and as a product, through the development of artifacts and new formations. Technology is based on scientific knowledge that seeks benefits for health conditions, aiming for empowerment and knowledge formation^(5)^.

These technologies can be divided into three categories: hard (represented by products), soft-hard (structured knowledge), and soft (a process of subjective relationships). Soft technologies are associated with the process of subjective relationships for implementing care, such as service management and the bond between professionals and patients. Soft-hards are characterized by how the professional applies their insights to deliver care, including seeking training and information on how to perform a specific intervention. On the other hand, hard technologies are primarily the use of products, instruments, and machines, including multiparametric monitors and software, as well as coverings used in wounds at various stages of healing^(6,7)^.

Consequently, technology in nursing is a process that involves different dimensions, resulting in a product, which can be a hard good, a theory, or a new way of doing something. Nursing care processes are often subjective and can produce intangible results. Therefore, the use of technologies for this purpose can also have such characteristics. Thus, technologies can be seen as a comprehensive concept that goes beyond the mere use of machines; it considers not only the product but also the result of work and a set of abstract actions that have a goal: healthcare^(5)^.

In this regard, there is a need to present the evidence that guides the care of burn patients in intensive care to provide essential information for the adoption of technologies that offer appropriate effectiveness, efficiency, and cost-effectiveness in the therapy of burn patients. Based on this premise, it is necessary to map the technologies discussed in national and international literature, particularly in recent years, with the aim of recognizing and integrating technological innovations into nursing care.

The findings from this study can support knowledge production in the field and assist nurses in organizing their actions through the use and integration of technologies in healthcare. The quality of nursing care, coupled with the use of technologies, can contribute to the prevention of complications and the increased survival of burn victims.

Moreover, this knowledge will add to the existing body of studies on this topic, with the distinct feature that this research aims to map the various types of technologies for burn treatment, especially in the ICU setting. It is worth noting that studies that gather information on various types of healthcare technologies in this specific context are not common.

Therefore, the guiding question of this study is: What evidence is available regarding the technologies adopted by the nursing team in the treatment of cutaneous burn injuries in ICU patients?

## OBJECTIVES

To analyze the technologies used by the nursing team in the treatment of skin lesions caused by burns in patients under intensive care.

## METHODS

### Ethical Aspects

Given that this is a scoping review, submission to the Research Ethics Committee was not required. However, measures were taken to ensure the reliability and accuracy of the information obtained from the selected publications.

### Study Design

The study is characterized as a scoping review, structured according to the recommendations of the Joanna Briggs Institute method, JBI Manual for Evidence Synthesis^(8)^, and based on the theoretical framework of Arksey and O’Malley^(9)^. It also follows the Preferred Reporting Items for Systematic Reviews and Meta-Analyses extension for Scoping Reviews (PRISMA-ScR) checklist^(10)^. The scoping review assessment checklist consists of 20 essential reporting items and 2 optional items to include when conducting a scoping review^(8)^.

### Methodological Procedures

The search strategy was executed in three phases, adhering to JBI guidelines^(11)^: Phase 1 involved identifying keywords and search terms, Phase 2 encompassed database searches, and Phase 3 entailed examining reference lists.

To formulate the research question, the PCC mnemonic was employed, which stands for Population, Concept, and Context^(11)^, as per the JBI Manual for Evidence Synthesis^(8)^, 2020 version. The study focused on burn victims in intensive care, with a particular emphasis on the technologies employed by the nursing team for the treatment of cutaneous burn injuries in an intensive care context.

The primary research question was: What evidence is available regarding the technologies adopted by the nursing team in the treatment of cutaneous burn injuries in patients in intensive care?

The study utilized Health Sciences Descriptors (DeCS), including Burn, Intensive Care Unit, Therapeutics, Technology, and Nursing Care. Medical Subject Headings (MeSH) were also incorporated, encompassing Burns, Intensive Care Units, Therapeutics, Technology, and Nursing Care. These descriptors were combined using the boolean operator AND. The selected databases for the search encompassed the Latin American and Caribbean Literature in Health Sciences (LILACS), Medical Literature Analysis and Retrieval System Online (MEDLINE), PubMed, Cumulative Index to Nursing and Allied Health Literature (CINAHL), accessed from July 2021 to October 2022 through the Virtual Health Library (BVS), COCHRANE, and the Portal of Journals of the Coordination for the Improvement of Higher Education Personnel (CAPES), employing the advanced search tool. Furthermore, a search in the reference lists of selected studies from the databases was carried out to identify and include new evidence in composing the sample for this review.

### Data Collection and Organization

To select studies and form the sample, the Boolean operator AND was used, as outlined in the search strategy: ((Burns) AND (Therapeutics)) AND (Technology), ((Burns) AND (Therapeutics)) AND (Intensive Care Units), ((Burns) AND (Nursing Care)) AND (Technology).

Inclusion criteria encompassed studies published in Portuguese, Spanish, or English, without any temporal restrictions. Grey literature, including articles and manuals on the topic cited in the references of selected studies, was included in the sample. Duplicate studies were excluded.

An adapted form recommended by the JBI^(11)^, was employed for data extraction to facilitate information synthesis. Variables extracted included publication details (title, month and year, authors, journal, and country of publication), the study’s objectives, methodological characteristics, primary results (types of technology used for cutaneous injury treatment), and the study’s level of evidence.

The classification of evidence levels (LE) was based on the model proposed by Galvão in 2006^(12)^, which comprises seven levels, with Level 1 indicating the highest level of evidence and Level 7 the lowest. These levels were categorized as follows: Level 1 - evidence from systematic reviews or meta-analyses of all relevant controlled randomized clinical trials or clinical practice guidelines derived from systematic reviews of controlled randomized clinical trials; Level 2 - evidence from at least one well-designed randomized controlled clinical trial; Level 3 - evidence from well-designed clinical trials that lack randomization; Level 4 - evidence from well-designed cohort and case-control studies; Level 5 - evidence from systematic reviews of descriptive and qualitative studies; Level 6 - evidence from a single descriptive or qualitative study; and Level 7 - evidence based on expert opinion and reports from expert committees.

### Data Analysis

Following the selection of articles, a thorough review of titles and abstracts was conducted, and the Rayyan software was used to facilitate the initial screening, ensuring alignment with the authors’ decisions regarding study inclusion or exclusion^(13)^. Data collection and analysis were performed by three independent reviewers, with consultation of a fourth reviewer in case of discrepancies. In the final stages, data extraction, information delimitation, and evidence analysis were conducted through a descriptive approach to characterize the studies.

This was carried out after analyzing the PRISMA-ScR flowchart. Data from the articles were incorporated into a tabular instrument prepared by the authors. This instrument included the publication’s characteristics (year, country of origin, title, and authors), study objectives, methodology, primary results (types of technology used for cutaneous injury treatment), and the study’s level of evidence. The findings were subsequently described and critically discussed to align with relevant literature, and the results were organized, presented in tables, and supported by existing literature.

## RESULTS

A total of 2,417 primary references were initially identified, and after applying the inclusion criteria, 751 full-text documents remained. Subsequently, titles and abstracts were reviewed, resulting in 48 studies for full-text examination, of which 14 aligned with the research objective.

**Figure 1:**
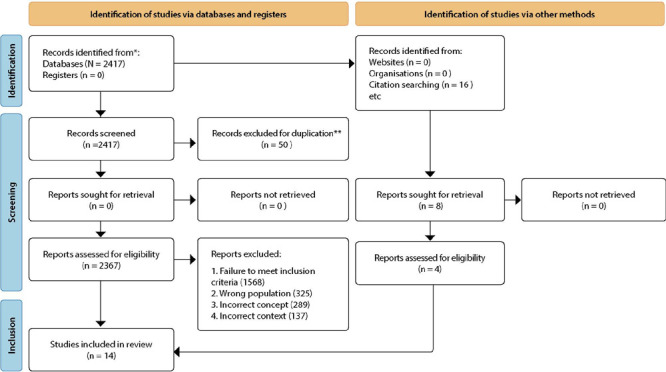
Flowchart illustrating the search and study selection process, adapted in accordance with PRISMA-ScR, Crato, Ceará, Brazil, 2022

To illustrate the article search and selection process, we employed an adapted flowchart based on the Preferred Reporting Items for Systematic Reviews and Meta-Analyses extension for Scoping Reviews (PRISMA-ScR)^(14)^, as depicted in Figure 1.

The sample for this review comprised 14 articles that discussed the technologies employed by the nursing team in the treatment of cutaneous burn injuries. Upon analyzing the data, it became apparent that burn treatment involves technologies for pain management, infection control, and wound care. The summary of the utilized studies is presented in Table 1, and the interventions are justified in Table 2. Please note that the identification code (ID) for each study will be denoted by the letter “S” (Study), followed by a sequential number, for instance: S1, S2, and so forth.

**Chart 1:** Summary table of the selected studies, Crato, Ceará, Brazil, 2022

ID	Title	Year/Country	Design	Interventions	LE
S1	*[Associação de membrana biológica de hemicelulose com pomada de estimulação da epitelização: Relato de caso]^(4)^*	2016/Brazil	Case study	Biological membrane of hemicellulose, associated with hydrolyzed collagen gel.	4
S2	Biobrane™ for burns of the pubic region: minimizing dressing changes^(15)^	2018/Singapore	Case report	Artificial skin.	4
S3	Care in patients with epidermal necrolysis in burn units. A nursing perspective^(16)^	2018/Spain	Descriptive cross-sectional study	Skin cleansing techniques, antiseptics, conservative skin management, and the use of artificial skin.	4
S4	Comparison of analgesic and anxiolytic effects of nitrous oxide in burn wound treatment^(17)^	2019/China	Randomized controlled clinical trial	Nitrous oxide (N2O) and 50% N2O.	1
S5	Effectiveness of a hydrogel dressing as an analgesic adjunct to first aid for the treatment of acute paediatric thermal burn injuries: study protocol for a randomised controlled trial^(18)^	2019/Australia	Randomized controlled clinical trial	Hydrogel.	1
S6	Enhanced wound-healing performance of a phytopolysaccharide-enriched dressing – a preclinical small and large animal study^(19)^	2017/China	Preclinical study	Calcium Alginate Polysaccharide Dressing (CAPS).	3
S7	Local Probiotic Therapy with Lactobacillus plantarum Mitigates Scar Formation in Rabbits after Burn Injury and Infection^(20)^	2017/United States of America	Experimental study	Lactobacillus plantarum.	2
S8	Randomized clinical trial of negative pressure wound therapy as an adjunctive treatment for small-area thermal burns in children^(21)^	2020/Australia	Randomized clinical trial	Silver dressing with silicone cover.	1
S9	*Synechococcus elongatus* PCC7942 secretes extracelular vesicles to accelerate cutaneous wound healing by promoting angiogenesis^(22)^	2019/China	Randomized controlled clinical trial	S. elongatus PCC 7942.	1
S10	New lupeol esters as active substances in the treatment of skin damage^(23)^	2019/United States of America	Randomized controlled clinical trial	Lupeol derivatives.	1
S11	Chitosan-Gentamicin Conjugate Hydrogel Promoting Skin Scald Repair^(24)^	2020/China	Randomized controlled clinical trial	Chitosan-gentamicin conjugate (CS-GT).	1
S12	*[Fator de crescimento transformador beta 3 e a melhora macro e microscópica de cicatrizes: Uma revisão bibliográfica]* ^(25)^	2019/Brazil	Literature review	Avotermin.	5
S13	*[Guia de prática clínica para o cuidado de enfermagem ao paciente queimado: Metodologia ADAPTE]^(26)^*	2021/Brazil	ADAPTE Methodology	3-layer nanocrystalline silver dressing; Hydrofiber with silver.	7
S14	*[Manual de queimaduras para estudantes]^(1)^*	2021/Brazil	Expert opinion – Manual	Pressotherapy; Collagenase; 1% silver sulfadiazine.	7

*ID - study identification code; Nº - Study number; LE - level of evidence.*

**Chart 2:** Characterization of technologies used in the treatment of cutaneous burn injuries, Crato, Ceará, Brazil, 2022

Category/Classification	Intervention Used	Justification
Antimicrobial and Healing / Hard Technology	*Lactobacillus plantarum*	Reduces the intensity and duration of Pseudomonal infection. Attenuates collagen deposition.
	Chitosan-gentamicin conjugate (CS-GT)	Promotes collagen fibrosis. Reduces cytokine levels in an inflammatory response. Accelerates wound healing.
	Silver hydrofiber	Facilitates healing and reduces the risk of wound bed infections. Provides bactericidal action against planktonic microorganisms.
	Collagenase	Promotes liquefaction of necrotic tissue without deteriorating granulation tissue. Restricts the formation of hypertrophic scars in superficial 2nd-degree burns. Reduces the basal substrate for bacterial proliferation.
Analgesic and Healing / Hard Technology	Artificial skin	Minimizes infection and promotes wound healing. Provides pain relief in epidermal necrolysis.
	Calcium alginate (CAPS)	Reduces wound size. Maintains moist wounds. Promotes granulation tissue growth, pain reduction, and scar formation. Absorbs wound fluids.
	Hemicellulose biological membrane	Pain relief. Accelerates the epithelialization process.
Analgésico/Tecnologia Dura	Hydrogel	Pain relief.
	Nitrous oxide (N2O) and 50% N2O	Reduces pain and anxiety.
Analgesic / Hard Technology	Nanocrystalline silver 3-layer	Minimizes wound trauma during dressing changes due to the low-adherence contact layer. Provides an effective antimicrobial barrier to the wound bed.
	Silver sulfadiazine 1%	Controls local infections by inhibiting bacterial growth. Indicated only in the early days of burn treatment, for necrotic tissue or infection.
Antimicrobial / Hard Technology	S. elongatus PCC7942 cyanobacterium	Accelerates cutaneous wound healing. Promotes angiogenesis and burn repair in mice.
	Silver hydroalginato	Favors angiogenesis. Accelerates tissue matrix maturation.
	Negative Pressure Wound Therapy (NPWT)	Favors spontaneous total reepithelialization. Reduces the risk of ischemic damage.
	Nile tilapia skin	Promotes tissue regeneration and healing capacity. Induces cell migration/division. Increases collagen synthesis by fibroblasts.
	Lupeol	Stimulates human skin cell proliferation.
	Avotermin	Influences fibroblast and myofibroblast migration to the wound site. Reduces excessive collagen in scar formation. Allows for greater migration of epithelial cells. Restores epithelial-mesenchymal interactions. Accelerates healing.
	Pressotherapy	Prevents the formation of hypertrophic or keloid scars. Reduces fibroblast proliferation and collagen synthesis.

The studies in this review were primarily conducted in China (n = 3), Brazil (n = 4), and Australia (n = 2). The United States of America, Singapore, and Spain each contributed one publication. Two articles did not specify their origin. In total, 10 studies were in English, and 2 were in Portuguese, published between 2016 and 2021.

Regarding the methodological design and the respective level of evidence, this review included: 6 randomized controlled clinical trials (LE = 1); 1 case study, 1 case report, and 1 descriptive cross-sectional study (LE = 4); 1 experimental study (LE = 2); 1 preclinical study (LE = 3), and 1 literature review (LE = 5). Subsequently, it was observed that level 1 evidence was the most prevalent (n = 6), while those with lower levels of evidence (5, 6, and 7) were either in the minority or not included in the sample findings. In other words, a significant number of articles included in this review possess a high level of evidence due to their study types.

Chart 2 provides an overview of the main technologies used in the treatment of burn victims, along with the type of intervention and the justification for using them, based on the 14 articles considered relevant in this study’s sample composition.

## DISCUSSION

Considering the analysis and synthesis of the results from this research, it was identified that the literature as a whole refers to the use of hard technologies in the care of burn patients in the ICU. However, it can be inferred that the use of soft- hard technologies is also present in the treatment of burn victims since, in some studies, there was a brief mention of the use of institutional protocols, manuals, or guidelines for the applicability of hard technologies. Soft technologies were not evident in the found studies. Taking these findings into account, it is crucial to emphasize that healthcare has historically been focused on hard and soft-hard technologies, and yet, soft technologies are fundamental, especially in this type of care since it is necessary to value human relationships and technical-scientific knowledge for welcoming and high-quality care^(7)^.

Regarding hard technologies, studies addressed the use of biological agents, such as the application of Lactobacillus plantarum, a probiotic therapy that led to the reduction in the intensity and duration of Pseudomonal infection. It was observed that the application of probiotic bacteria, even in the absence of a superinfectious pathogen, attenuates collagen deposition stimulated by burns^(20)^. Additionally, another study with a biological agent discussed the application of the cyanobacterium S. elongatus PCC7942, where the bacterium secretes extracellular vesicles to accelerate the healing of cutaneous wounds, promoting angiogenesis and burn repair in mice^(22)^.

Several references in this sample discuss the use of artificial skin, especially for patients undergoing general anesthesia for burns in other parts of the body. The successful application of this biosynthetic dressing minimized infection and promoted wound healing for partial-thickness burns of the pubic region, requiring the combination of the dressing with the removal of pubic hair and the insertion of a urinary catheter to provide complete wound coverage^(15)^. In another study, the benefits of using this dressing in patients with epidermal necrolysis, such as pain reduction, a decrease in dressing change frequency and time, and cost reduction benefits, were mentioned^(18)^.

Hydrogel dressings provide pain relief through an evaporative cooling effect, create a moist environment that soothes exposed nerve endings on the skin, and dissipate heat from the wound. They come in a wide variety of sizes that can be applied to all areas of the body. In a randomized clinical trial, participants had hydrogel applied after initial burn first aid for a minimum of 20 minutes, followed by standard burn care. Favorable results were confirmed through pain scales, vital sign assessments, patient behavior observations, and other parameters^(18)^.

In the case study conducted by Lopes et al., rapid epithelialization occurred due to the use of ointment that stimulates epithelialization, along with the use of hemicellulose biological membrane, which is considered a product that promotes a range of expected therapeutic properties in burn wound management, also assisting in occluding nerve endings, reducing the pain from second-degree burns. The use of skin regeneration ointments occurs in the final stage of treatment or when there are no cavities to be granulated; they stimulate the epithelialization process. In the study, there was effective scar tissue formation in just five days, without major injuries to the patient and without complications associated with burn trauma^(4)^.

Wang et al. evaluated the efficacy of Calcium Alginate (CAPS) in a third-degree burn model in pigs and rats, especially in relation to pain, inflammatory cytokines, and scar formation. Alginate has been widely used and tested due to its biocompatibility, biodegradability, relatively low cost, low toxicity, and gelation properties. The evidence also indicated that topical treatment with alginate for skin defects is effective in reducing wound size. This dressing keeps wounds moist and promotes granulation tissue growth and resulting epidermization, reducing pain and scar formation. Additionally, dry alginate dressings absorb wound fluids through ion exchange and gel without disintegrating^(19)^.

Oliveira and Peripato also mention technologies such as silver-associated hydroalginate, which has antimicrobial activity and reduces exudate. They also discuss chitosan gels combined with silver sulfadiazine, which promote angiogenesis. In addition to dressings, topical agents for superficial burns, and grafts in severe cases, the authors provide information about negative pressure wound therapy (NPWT) with dermal regeneration matrix. This dressing can accelerate tissue matrix maturation time to 14.57 days and activates cell granulation^(27)^.

Papain is a widely used technology for wounds of various etiologies. It is used as a debriding agent and has anti-inflammatory properties. Papain acts at the wound edges, stimulating cell reproduction and inhibiting microorganism growth. In addition to its numerous benefits at the wound bed, papain is cost-effective compared to other types of commercial dressings. This technology can be used in various concentrations: 2% for granulation tissue, 4 to 6% for liquefaction necrosis, and 8 to 10% for coagulation necrosis^(28)^.

A randomized clinical trial mentioned in this review compared the effects of nitrous oxide (N2O) titration and 50% N2O in the treatment of burns for pain and anxiety relief. N2O is a sedative and anxiolytic that is considered safe, simple, and effective analgesia for burn patients. The trial found that N2O titration is a more appropriate intervention as analgesia during burn dressing than exposure to 50% N2O. However, further studies are needed to identify the analgesic effects in all age groups and to develop guidelines and operational procedures^(17)^.

Negative pressure wound therapy (NPWT) is a type of active wound treatment that promotes healing in a moist environment through controlled locally applied subatmospheric pressure. A randomized clinical trial with children with acute thermal burns covering less than 5% of their total body surface area showed that 96 out of 101 participants (95%) achieved total spontaneous reepithelialization^(21)^.

Furthermore, the average time for reepithelialization was 10 days in the control group and 8 days in the NPWT group. The expected 22% reduction in wound closure time corresponded to a reduction in the number of dressing changes required. The intervention was performed using a vacuum pump set to continuous subatmospheric pressure of 80 mmHg. For burns involving the extremities in children under 12 months, a pressure of 40 mmHg was applied to reduce the risk of ischemic damage^(21)^.

Lima-Júnior et al. discussed biological dressings, with the most well-known being Nile tilapia skin (Oreochromis niloticus). This method is used because fish skin has a similar microscopic and morphological structure to human skin, as well as the ability to regenerate and heal tissues. It influences wound healing by adhering well to the burn, retaining moisture, and regenerating injured tissue. Additionally, the biomolecules it contains have the potential to induce cell migration/division and increase collagen synthesis by fibroblasts^(29,30)^.

Chitosan-gentamicin conjugate (CS-GT) has efficient antimicrobial properties, as evidenced by viability assays and L929 cell hemolysis, which showed that CS-GT is non-cytotoxic, with good cytocompatibility and hemocompatibility. CS-GT hydrogel was prepared using CS-GT as an active repair component and PVP as a matrix. The application of this hydrogel accelerated wound healing and shortened healing time in animals. Biochemical analysis showed that the CS-GT hydrogel promoted collagen fibrosis and reduced cytokine levels in an inflammatory response by facilitating total protein and hydroxyproline synthesis in granulation tissue, thereby accelerating wound healing. All these results demonstrate that the CS-GT hydrogel is a promising and innovative burn repair dressing^(24)^.

A research study involving derivatives of the triterpene lupeol demonstrates that isonicotinate of lupeol, acetate, and propionate stimulate the proliferation of human skin cells, resulting in an increase of over 30% in cell concentration compared to control samples. These lupeol esters show promising activity in stimulating skin repair processes, suggesting that they can be applied as active substances in topical formulations, particularly in the treatment of cutaneous burns^(23)^.

Avotermina, which is the recombinant human transforming growth factor beta 3 (TGF-ß3), has been shown to be effective in promoting epidermal restoration and organization similar to the original in both the papillary and reticular dermis. Its intradermal application significantly accelerated scar erythema reduction. TGF-ß3 has a positive impact on various stages of the healing process, including the migration of fibroblasts and myofibroblasts to the wound site. Furthermore, it stimulates increased expression of molecules involved in extracellular matrix remodeling (metal-loproteinases), reducing excess collagen. It also facilitates the migration of epithelial cells, accelerating the restoration of epithelial-mesenchymal interactions, angiogenesis, and scar maturation^(25)^.

The technology of Three-layer Nanocrystalline Silver, which consists of a dressing with an absorbent central core and outer layers of low-adherence polyethylene mesh coated with nano-crystalline silver, is effective as an antimicrobial barrier for partial and full-thickness wounds, including burns. It can treat a variety of conditions, such as pressure ulcers, venous ulcers resulting from diabetes, and infected wounds^(26)^.

Hydrofiber with Silver is a technology that converts exudate into gel when it comes into contact with the dressing, facilitating the healing process and reducing the risk of infections within the wound bed. The silver present in this dressing offers bactericidal properties, affecting both planktonic microorganisms and those that form biofilms. It is recommended for dermal thickness burns, moderately exudative wounds, infected wounds, or those at high risk of infection and/or bleeding^(26)^.

Pressotherapy involves the use of compression garments on burn victims as a prophylactic approach to prevent the development of hypertrophic or keloid scars. The application of pressure restricts microcirculation, reducing fibroblast proliferation and collagen synthesis. The ideal pressure should be between 20 and 30 mmHg, exceeding capillary pressure without reducing peripheral vascularization^(1)^.

1% Silver Sulfadiazine is an effective topical technology for controlling local infections. It acts as a competitive agonist of para-aminobenzoic acid (PABA), inhibiting bacterial growth. Silver sulfa-diazine is effective against gram-negative bacteria and the fungus Candida albicans. However, prolonged use can impair healing and is recommended only in the early days of burn treatment, in necrotic tissues, or when there is suspicion of infection^(1)^.

Collagenase is an ointment containing enzymes derived from Clostridium histolyticum that break the peptide bonds of collagen. This enzymatic action involves the hydrolysis of protein bonds and the breaking of the triple-helix structure of collagen. This allows the liquefaction of necrotic tissue without harming granulation tissue, keeping viable cells alive and contributing to angiogenesis and epithelialization. However, collagenase should not be used in combination with heavy metals like silver, as they inactivate the enzyme^(1)^.

### Study limitations

In some of the studies included in this review, comprehensive details about the evaluated technologies were lacking. This limited our ability to conduct a thorough and comprehensive analysis of certain interventions. The absence of detailed information regarding the specific features of the technologies used, such as their compositions, application methods, and dosages, can impact the accuracy of the conclusions. Additionally, in some databases, the use of controlled descriptors was absent, which may have led to some information loss.

### Contributions to the Nursing, Healthcare, or Public Policy Field

The results obtained provide a solid foundation for discussions regarding the technologies utilized by nursing teams in the treatment of cutaneous injuries resulting from burns in intensive care patients. Consequently, this contributes to evidence-based knowledge development in this field, promoting more systematic and high-quality nursing care for this specific population.

## FINAL CONSIDERATIONS

This mapping has allowed for the identification of the technologies most frequently employed by nursing teams in the treatment of cutaneous burn injuries in intensive care patients. This information holds great significance in supporting nursing practice.

Furthermore, based on the analyzed evidence, we can conclude that technologies play a crucial role in the treatment of cutaneous burn injuries. This is particularly relevant because burns can lead to adverse outcomes, such as infections in the burned areas, which may progress to sepsis and multiple organ dysfunction, being one of the main causes of mortality in burn patients.

The reviewed studies presented various technologies applicable to burn treatment, including different types of wound dressings and coverings, biological agents, and non-pharmacological therapies for pain relief and infection control. These therapeutic interventions contribute to the regeneration of burned tissues and provide comfort to patients. However, it is essential to emphasize that there is no single standardized approach to nursing care in burn treatment, and the choice of the appropriate technology depends on the burn assessment, local conditions, and cost-effectiveness.

Nonetheless, we observed a lack of mention of “soft” technologies, such as the importance of interpersonal and professional communication, creativity, relationship building, service management, and patient-centered care. This underscores the need for further research addressing this theme and highlighting the various healthcare technologies to support clinical nursing practice in caring for burn victims in intensive care.
